# Association of age-related hearing loss with cognitive impairment and dementia: an umbrella review

**DOI:** 10.3389/fnagi.2023.1241224

**Published:** 2023-09-18

**Authors:** Guo Ying, Guangran Zhao, Xianpeng Xu, Su Su, Xin Xie

**Affiliations:** ^1^Department of Acupuncture and Moxibustion, The Second Affiliated Hospital of Heilongjiang University of Chinese Medicine, Harbin, China; ^2^Department of Otolaryngology, Hospital of Chengdu University of Traditional Chinese Medicine, Chengdu, China; ^3^Department of Acupuncture and Moxibustion, Zhuhai Hospital of Integrated of Traditional Chinese Medicine and Western Medicine, Zhuhai, China; ^4^Department of Rehabilitation, Heilongjiang Provincial Hospital, Harbin, China

**Keywords:** age-related hearing loss, cognitive impairment, dementia, umbrella review, Alzheimer’s disease

## Abstract

**Background:**

Hearing loss, cognitive impairment and dementia have become common problems for older adults. Currently, systematic reviews and meta-analyses of the association between age-related hearing loss (ARHL) with cognitive impairment and dementia may have inconsistent results. To explore and validate the association between ARHL with cognitive impairment and dementia through summarizing and evaluating existing evidence.

**Methods:**

From inception to February 01, 2023, PubMed, Web of Science, Embase, and Cochrane Library databases were systematically searched. AMSTAR 2 was used to evaluate methodological quality and GRADE system was used to evaluate evidence quality. We summarized the basic characteristics of the included studies and extracted effect data for ARHL with cognitive impairment and dementia. Forest plots were used to describe the relative risk associated with ARHL and cognitive impairment, and the relative risk associated with ARHL and dementia, respectively.

**Results:**

A total of 11 systematic reviews and meta-analyses met the inclusion criteria. Overall, the methodological quality of the included SRs/MAs was moderate and the quality of the evidence was low. The combined results found that the pooled risk ratio of ARHL and cognitive impairment was 1.30 (random-effects; 95% CI 1.16 to 1.45), and the pooled risk ratio of ARHL and dementia was 1.59 (random-effects; 95% CI 1.34 to 1.90).

**Conclusion:**

Based on the evidence reported in this umbrella review, age-related hearing loss is significantly associated with cognitive impairment and dementia. Hearing loss may be a high risk factor for cognitive impairment and dementia in older adults.

## 1. Introduction

Age-related hearing loss (ARHL) is a prevalent complex sensory deficit among older adults, resulting from the cumulative effects of aging on the auditory system (Bowl and Dawson, 2019). The deprivation of hearing in older adults due to ARHL could distance them from regular social activities, potentially leading to social isolation, loneliness, cognitive impairment, and an elevated risk of frailty and falls (Kamil et al., 2016; Rutherford et al., 2018). The count of individuals with hearing loss, previously estimated at 1.57 billion, is projected to surge due to global population aging and demographic shifts (GBD 2019 Hearing Loss Collaborators, 2021). According to the World Health Organization’s World Report on Hearing, approximately 2.5 billion people worldwide could live with varying levels of hearing loss by 2050, with approximately 700 million individuals requiring treatment or rehabilitation services (Chadha et al., 2021). Hearing loss is widespread among older age groups, with those >50 years constituting 62.1% of all hearing loss cases (GBD 2019 Hearing Loss Collaborators, 2021). The risk of ARHL escalates with age, with an estimated prevalence of approximately 40% in the population > 65 years and 50–80% in those >80 years (Gates and Mills, 2005; Davis et al., 2016). Patients experiencing cognitive decline struggle to perceive and process target speech amidst background noise or competing speech, a challenge that might manifest several years before the onset of dementia. Therefore, early identification of hearing loss is pivotal in effectively preventing cognitive impairment and dementia among older adults.

ARHL, ranked the third most formidable chronic disability among older adults, demonstrates its potential relationship with cognitive impairment and dementia (Jafari et al., 2019). Despite evidence indicating ARHL as a possible risk factor for cognitive impairment and dementia, the underlying mechanism remains unclear (Chern and Golub, 2019). As research has deepened, the hypothesis proposing a causal relationship between ARHL and cognitive impairment has gained widespread attention recently. The support for the involvement of neurodegeneration in both ARHL and cognitive impairment comes from research conducted on older adults’ perception and cognition abilities (Fischer et al., 2016). This hypothesis attributes the simultaneous occurrence of ARHL and cognitive impairment to brain atrophy and biological decline (Lalwani et al., 2019). The information-degradation theory states that compromised peripheral auditory function diminishes speech quality due to environmental noise or hearing loss, requiring more “auditory effort” to process acoustic signals. Consequently, limited cognitive resources are diverted from cognitive to hearing tasks, precipitating cognitive decline (Humes et al., 2013). Moreover, the sensory deprivation hypothesis, similar to the information-degradation theory, emphasizes how sensory deprivation prompts compensatory cortical reorganization and neural changes that hinder regular auditory perception and cognitive function (Slade et al., 2020). Although research into the ARHL-cognition relationship is extensive, the absence of robust evidence still clouds the causal relationship between the two, necessitating further clarification of their connection (Livingston et al., 2020).

## 2. Methods

This umbrella review compiles evidence from systematic reviews/meta-analyses (SRs/MAs) concerning multiple clinical questions. It was conducted per a pre-established protocol and adhered strictly to the Preferred Reporting Items for Systematic Reviews and Meta-Analyses (PRISMA) reporting guidelines (Liberati et al., 2009). The protocol was registered in PROSPERO (registration number CRD42022372393).

### 2.1. Search strategy and selection criteria

Eligible articles were collected through a literature search and screened by two investigators (GRZ, YG) for their title, abstract, and full-text relevance. Databases, including PubMed, Web of Science, Embase, and Cochrane Library, were searched from inception to February 01, 2023. Medical Main Headings (MeSH) terms and keywords encompassed concepts such as “presbycusis” or “age-related hearing loss,” “cognition,” “cognition disorders” or “cognitive dysfunction,” “dementia” or “Alzheimer’s disease,” “meta-analysis” or “systematic review.” Moreover, MeSH and keywords were expanded to include “hearing” or “hearing loss,” and “aged” ensuring comprehensive coverage. References of selected articles were manually reviewed to avoid omitting potentially eligible articles. The definitions of cognitive impairment, dementia, and Alzheimer’s disease are provided in Appendix (p. 2).

This review exclusively incorporated SRs/MAs analyzing the relationship of ARHL with cognitive impairment and dementia. Encompassing cohort, cross-sectional, prospective, and observational studies, the included articles were required to meet specific criteria: (1) The baseline participant population consisted of older adults with community health or cognitive impairment (or dementia) who underwent hearing assessments. Particular groups (such as those with coronary heart disease or hearing-affecting conditions) were excluded; (2) Interventions included peripheral and central hearing loss assessed using diverse methods, including pure tone audiometry, speech audiometry, auditory evoked potentials, self-reported hearing loss, and other primary hearing assessment methods. Cognition was assessed with commonly used tests, such as the Mini-Mental State Examination (MMSE), Montreal Cognitive Assessment (MoCA), Alzheimer’s Disease Assessment Scale (ADAS), and criteria from the National Institute of Neurological and Communicative Disorders and Stroke and the Alzheimer’s Disease and Related Disorders Association (NINCDS-ADRDA). (3) Comparison of cognitive function between patients with and without hearing loss. (4) Outcomes included incidence or prevalence of cognitive impairment or dementia in individuals with hearing loss compared to those with normal hearing and investigations into the potential relationship or risk between hearing loss and cognition. The exclusion criteria were as follows: (1) The title, abstract, and full text of the article were published in a non-English language; (2) Original studies, case reports, conference papers, guidelines, posters, letters, graduate dissertations, or duplicates.

### 2.2. Methodological and evidence quality evaluation

Methodological and evidence quality evaluations were independently conducted by two investigators (GRZ, YG) using the AMSTAR 2 tool (Shea et al., 2017; A Measurement Tool to Assess Systematic Reviews 2) and the GRADE system (Guyatt et al., 2008; Grading of Recommendations Assessment, Development, and Evaluation), respectively. Any disagreements were resolved through mutual consultation, referencing authoritative guidelines, or consulting a third experienced professor.

The AMSTAR 2 was utilized to assess the methodological quality of the included SRs/MAs and to rate the overall study quality. Comprising 16 evaluation items, each corresponding to a standardization question, the AMSTAR 2 emphasizes the importance of critical things that are pivotal in producing systematic reviews and the validity of results. To prevent masking serious methodological shortcomings due to high overall scores, the AMSTAR 2 R&D team recommends focusing on seven critical items: 2, 4, 7, 9, 11, 13, and 15. Each item was evaluated as “yes,” “partly yes,” or “no” based on the degree of satisfaction. The quality level of system evaluation was categorized as high, moderate, low, and very low based on the results of these evaluations.

The GRADE system pertains to evidence-quality grading and recommendation strength across clinical questions, study design, and outcome indicators. It defines evidence quality and recommendation strength, primarily evaluating the evidence quality grade of system evaluation. This approach surpasses the limitation of considering evidence quality solely from the research design perspective. The GRADE system classifies evidence quality into four levels: high, moderate, low, and very low, aiming for transparency and simplicity. Although the GRADE system automatically downgrades the strength of evidence for observational studies, three criteria that enhance evidence quality are particularly applicable to such analyses. When the effect size of observational studies is substantial, it is feasible to elevate evidence by one or even two notches (Kien et al., 2013). Assessing evidence quality for systematic reviews involves five downgrade factors and three upgrade factors. Downgrade factors encompass risk of bias, inconsistency in results, indirectness of evidence, imprecision, and publication bias. Upgrade factors include a substantial effect, a potential confounder that could alter it, and dose–response gradients.

### 2.3. Data extraction and statistical analysis

Data extraction was carried out independently by two investigators that fulfilled the inclusion criteria. In instances of disagreement, a third expert mediated and finalized the decisions to ensure consensus. Extracted data encompassed fundamental characteristics of each qualified SRs/MAs, including author, publication year, country, number of studies included, participant count, quality assessment, design, outcomes, main conclusions, effect size with 95% confidence interval (CI), *p* value, and *I*^2^ value.

When available, aggregate effects, CIs, and heterogeneity data from the meta-analysis were extracted for descriptive analysis. We summarized the effect sizes regarding the relationships of hearing loss with cognitive impairment and dementia. Forest plots illustrated these relationships. Statistical analysis was conducted utilizing a random-effects model for the combined study and separately for cross-sectional and cohort studies. Subgroup data analysis was performed with adequate data, using Stata version 17 to generate forest plots.

### 2.4. Overlap evaluation

Recent years have seen a rise in overlapping studies within systematic reviews, addressing the potential double counting of the same research across two or more reviews. Ignoring or improperly assessing overlapping assessments could notably affect qualitative analysis or statistical weighting. Among numerous methods for evaluating overlap in umbrella overview development, the Graphical Representation of Overlap for Overviews (GROOVE) tool is widely considered the most comprehensive and user-friendly option (Pérez-Bracchiglione et al., 2022). Matrices of evidence and the calculation of corrected covered area (CCA) constitute part of GROOVE’s overlap measurement approach. These matrices visually depict overlap relationships among SRs/MAs, while CCA derives variables from the evidence matrix and computes the overlap rate using a specific formula.

## 3. Results

### 3.1. Results of literature search

**Figure 1:**
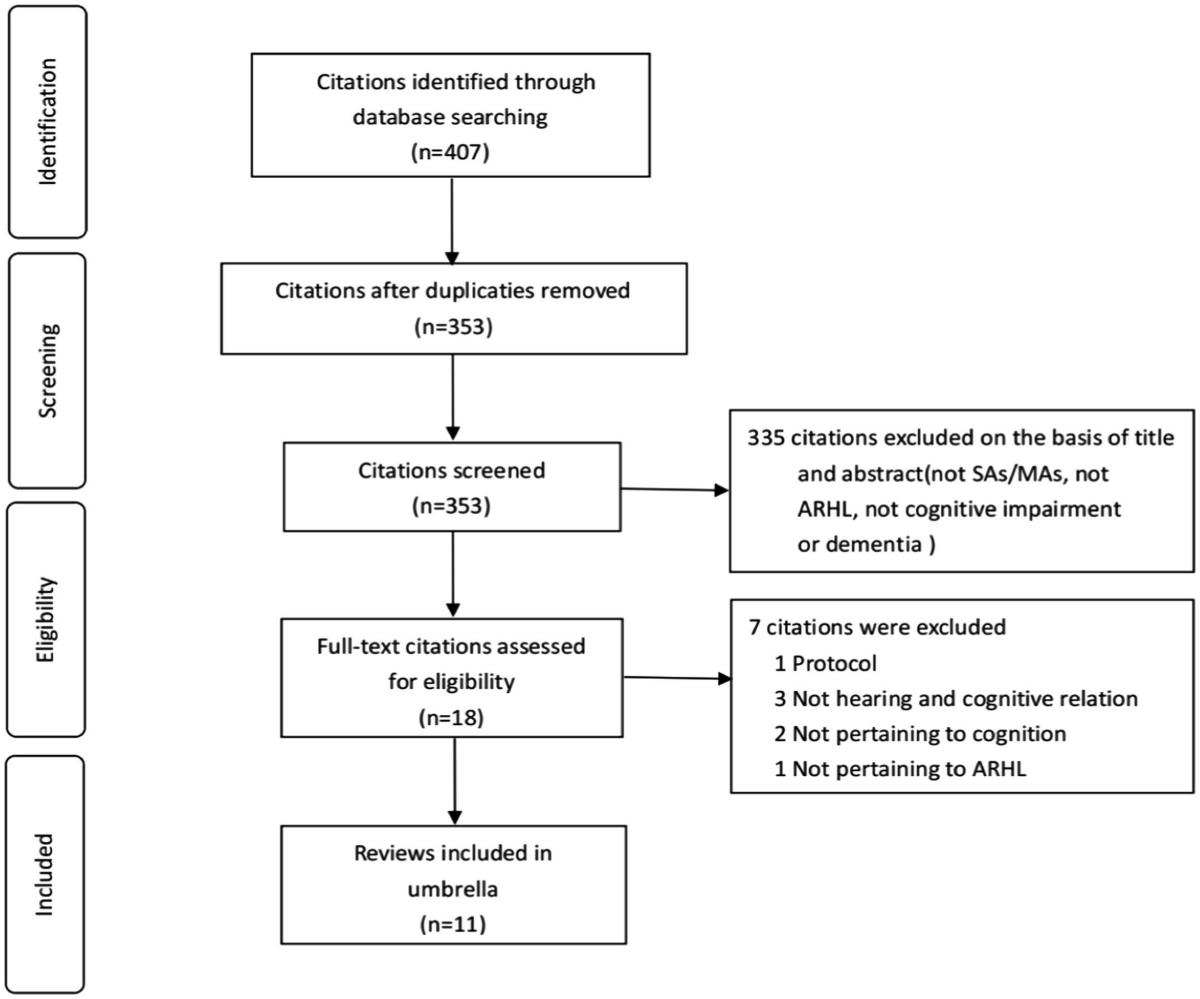
Flow diagram of systematic review selection.

An initial search across four electronic databases yielded 407 citations related to ARHL, cognitive impairment, and dementia. After removing 54 duplicates, we screened 353 citations by title and abstract, retaining 18 for further assessment. In our umbrella review, subsequent careful full-text screening included 11 sources (Thomson et al., 2017; Wei et al., 2017; Zheng et al., 2017; Ford et al., 2018; Loughrey et al., 2018; Yuan et al., 2018; Utoomprurkporn et al., 2020; Völter et al., 2020; Liang et al., 2021; Kwok et al., 2022; Lau et al., 2022). Figure 1 illustrates the flow chart of literature screening.

### 3.2. Characteristics of the included studies

The 11 systematic reviews and meta-analyses included in this study were published between 2016 and 2021. Of these, four were from European authors, three from American, three from Chinese, and one from Australian. The quality assessment methods used varied among the included studies: six studies employed the Newcastle-Ottawa Scale (NOS), four did not specify any quality assessment method, and one used the Strengthening the Reporting of Observational Studies in Epidemiology (STROBE). The topics covered in the reviews included the correlation between ARHL and cognitive impairment (7 studies), the correlation between ARHL and dementia (4 studies), and the correlation between ARHL and Alzheimer’s disease (3 studies). Further details can be found in Table 1.

**Table 1:** Characteristics of the included studies.

Author(s); year	Country	No. of studies	No. of subjects	Quality assessment	Main conclusions	Design/outcomes	Relative effect (95% CI)	*p*-value	*I* ^2^
Lau et al. (2022)	UK	34	48,017	NOS	Significant association between hearing loss and mild cognitive impairment	CS/CI	RR = 1.44 (1.27, 1.64)	<0.0001	0%
Loughrey et al. (2018)	Ireland	40	34,471	STROBE	Age-related hearing loss may be a biomarker and modifiable risk factor for cognitive decline, cognitive impairment, and dementia	CS/CI	OR = 2.00 (1.39, 2.89)	<0.001	83.1%
						CO/CI	OR = 1.22 (1.09, 1.36)	<0.001	0%
						CS/Dementia +AD	OR = 2.42 (1.24, 4.72)	0.01	0%
						CO/Dementia	OR = 1.28 (1.02, 1.59)	0.03	69.7%
						CO/AD	OR = 1.69 (0.72, 4.00)	0.23	72.6%
Zheng et al. (2017)	Chinese	4	7,461	NOS	Hearing impairment significantly increased the risk of cognitive impairment and was not significantly different from the risk of Alzheimer’s disease	CO/AD	RR = 4.87 (0.90, 26.35)	0.066	94.5%
						CO/CI + AD	RR = 2.82 (1.47, 5.42)	0.002	92.4%
Ford et al. (2018)	Australia	14	227,614	/	Hearing impairment leads to increased risk of dementia	CO/Dementia	HR = 1.49 (1.30, 1.67)	0.010	53%
Thomson et al. (2017)	America	17	1,378,444	/	Hearing loss is associated with a higher incidence of dementia in older adults	/	/	/	/
Utoomprurkporn et al. (2020)	UK	12	950	/	MoCA scores were significantly lower in people with hearing loss than in people with normal hearing	NS/CI(MoCA)	MD = -1.66 (−2.74, −0.58)	0.003	78%
Völter et al. (2020)	Germany	4	425	/	Subjects with hearing loss had lower MMSE and MoCA scores than subjects with normal hearing	NS/CI(MMSE, MoCA)	SD = 0.47 (2.01, 3.86)	<0.0005	/
Kwok et al. (2022)	America	6	393	NOS	The meta-analysis showed that the mean hearing threshold (dB HL) was higher in the AD cohort than in the normal cohort	CO/AD(0.5–2 kHz PTA)	MD = 2.40 (0.75, 4.05)	0.00044	/
						CO/AD(0.5–2 kHz PTA)	MD = 3.12 (1.60, 4,64)	0.0001	/
Yuan et al. (2018)	Chinese	11	176,893	NOS	Older adults have higher levels of hearing loss and a correspondingly higher risk of cognitive impairment	CO/CI(follow-up≤6 years)	RR = 1.29 (1.04, 1.59)	0.02	0%
						CO/CI(follow-up>6 years)	RR = 1.57 (1.13, 2.20)	0.008	0%
						CO/CI(total)	RR = 1.36 (1.14, 1.63)	0.0007	0%
Wei et al. (2017)	America	10	15,521	NOS	Hearing impairment has been linked to a higher risk of mild cognitive impairment and dementia in older adults	CO/CI	RR = 1.30 (1.12, 1.51)	0.411	0%
						CO/Dementia	RR = 2.39 (1.58, 3.61)	0.000	81.4%
Liang et al. (2021)	Chinese	14	726,900	NOS	Hearing loss may be an independent risk factor for dementia and AD	CO/Dementia	HR = 1.59 (1.37, 1.86)	<0.001	86%
						CO/AD	HR = 2.24 (1.32, 3.79)	0.003	2%

NOS, Newcastle–Ottawa Scale; STROBE, Strengthening the Reporting of Observational Studies in Epidemiology; MoCA, Montreal Cognitive Assessment; MMSE, Mini-Mental State Examination; PTA, Pure-tone audiogram; CS, Cross-sectional; CO, Cohort; NS, Not Stated; AD, Alzheimer’s Disease; CI, Cognitive Impairment; RR, Relative Risk; OR, Odd Ratio; HR, Hazard Ratio; SD, Standard Difference; MD, Mean Difference.

### 3.3. Methodological and evidence quality

AMSTAR 2 was employed to assess the methodological quality of the included SRs/MAs. Among the 11 studies, one (9.1%) was rated as high quality, seven (63.6%) as moderate quality, one (9.1%) as low quality, and two (18.2%) as very low quality. Despite their well-developed nature, several studies showed methodological shortcomings, such as incomplete search strategies. However, offering entirely favorable remarks within the search strategy section is challenging due to the absence of gray literature searches, search registration, consultation with field experts, and a renewed search within 24 months after review completion. Another common factor impacting methodology quality was the author’s failure to establish a transparent review methodology in advance and provide an exclusion list and reasons, leading to a reduction in methodological rigor. Analysis revealed that the decision of whether to address publication bias played a pivotal role in the decline of methodological quality, with only two studies examining this factor. Appendix (p. 3) provides more detailed evaluation of AMSTAR 2 projects.

The GRADE system evaluated evidence quality in all included SRs/MAs. Among the 21 evaluation items derived from the 11 reviews, only one (4.8%) was assessed as moderate quality, 11 (52.3%) as low quality, and nine (42.9%) as very low quality. The inherent classification of observational studies as low quality in the GRADE system contributed to the overall low evidence quality. Despite these shortcomings of observational studies, we acknowledge the system’s assessment. Although the overall evidence quality of evidence is not encouraging, only 19 out of 105 downgrade factor evaluations received a “−1,” and six strict upgrade factors received a “1.” Appendix (p. 4) provides a more detailed evaluation of the downgrade factor and upgrade factors of the GRADE system.

### 3.4. Overlap between included reviews

**Figure 2:**
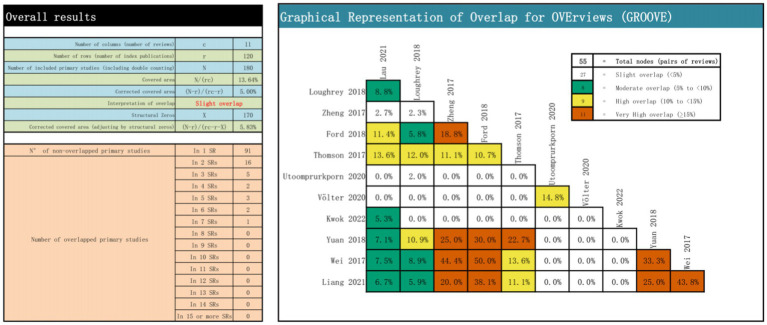
Overlapping of the included reviews.

A total of 55 overlapping associations were identified in the 11 reviews. Among these, 27 nodes had slight overlap, eight had moderate overlap, nine had high overlap, and 11 had very low overlap. A quantitative analysis using CCA indicated an overall CCA of 5.00%, categorizing the overlap as slight. For a comprehensive visual representation, refer to Figure 2, which includes the CCA formula components, calculation, overlapping and non-overlapping primary study counts, and the results presented at each node.

### 3.5. Results of systematic reviews

#### 3.5.1. Association between ARHL and cognitive impairment

**Figure 3:**
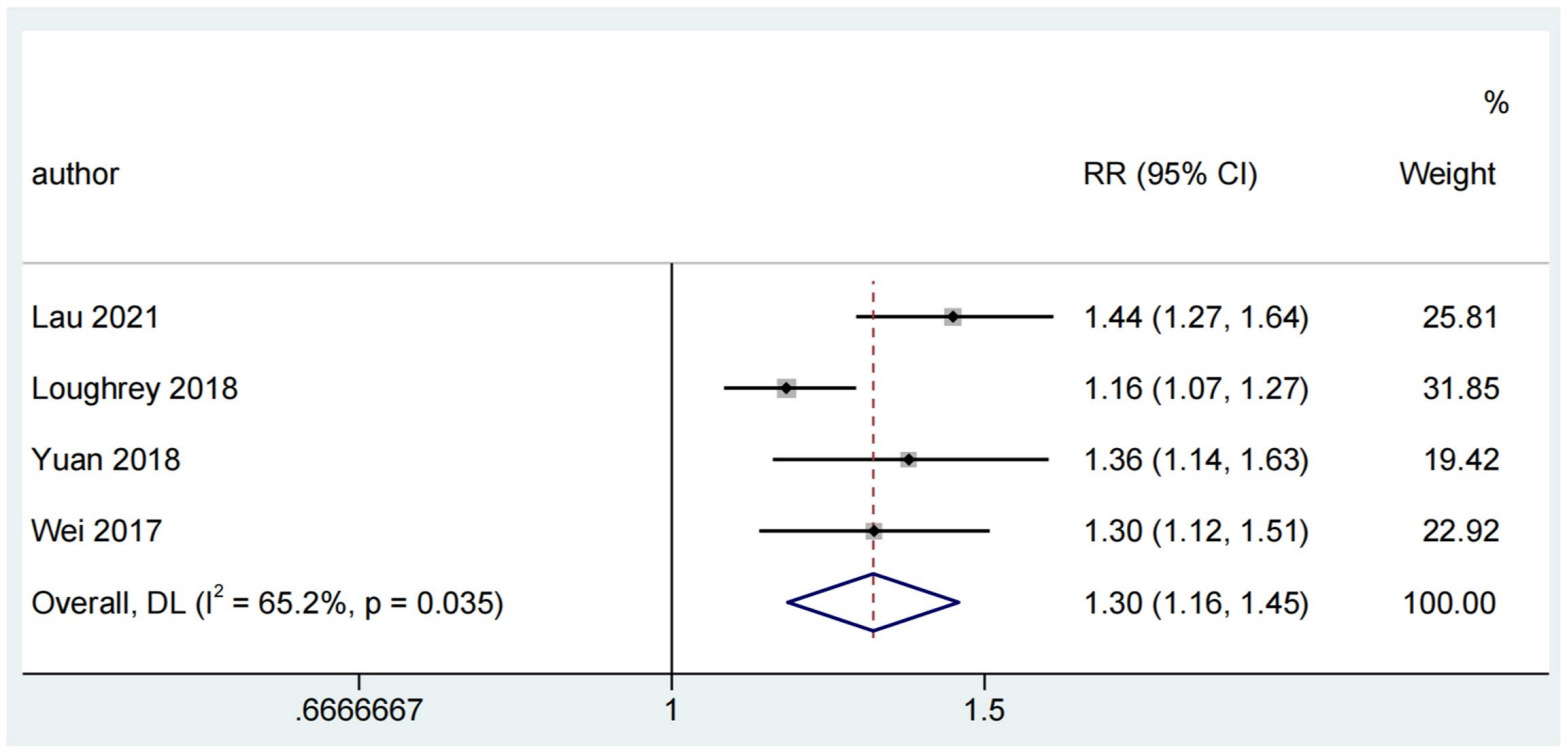
Forest plot of the association between ARHL and risk of cognitive impairment.

The association between ARHL and cognitive impairment was examined in six reviews (Wei et al., 2017; Loughrey et al., 2018; Yuan et al., 2018; Utoomprurkporn et al., 2020; Völter et al., 2020; Lau et al., 2022), of which four (Wei et al., 2017; Loughrey et al., 2018; Yuan et al., 2018; Lau et al., 2022) were used for the pooled analysis. The combined risk ratio for ARHL and cognitive impairment was 1.30 (random-effects; 95% CI: 1.16–1.45; *p* = 0.035; *I*^2^ = 65.2%). The forest plot (Figure 3) shows the results of the combined meta-analysis for ARHL and cognitive impairment. All the reviews indicated a significant association between ARHL and cognitive impairment. In a study (Loughrey et al., 2018) encompassing both cross-sectional and cohort studies, a substantial association between ARHL and cognitive impairment was identified. This study also revealed significant heterogeneity (Q range, 0.1 to 23.7) and considerable inconsistencies evident in cross-sectional studies, though not in cohort studies. Another meta-analysis (Utoomprurkporn et al., 2020) highlighted worse MoCA scores for individuals with hearing loss, with a pooled mean difference of −1.66 (95% CI: −2.74 to −0.58) from those with normal hearing. Furthermore, a distinct review (Völter et al., 2020) indicated that MoCA and MMSE scores were 2.94 points (SD = 0.47; 95% CI: 2.01–3.86) lower than those with normal hearing, with a significant difference (*p* < 0.0005).

#### 3.5.2. Association between ARHL with dementia and Alzheimer’s disease

**Figure 4:**
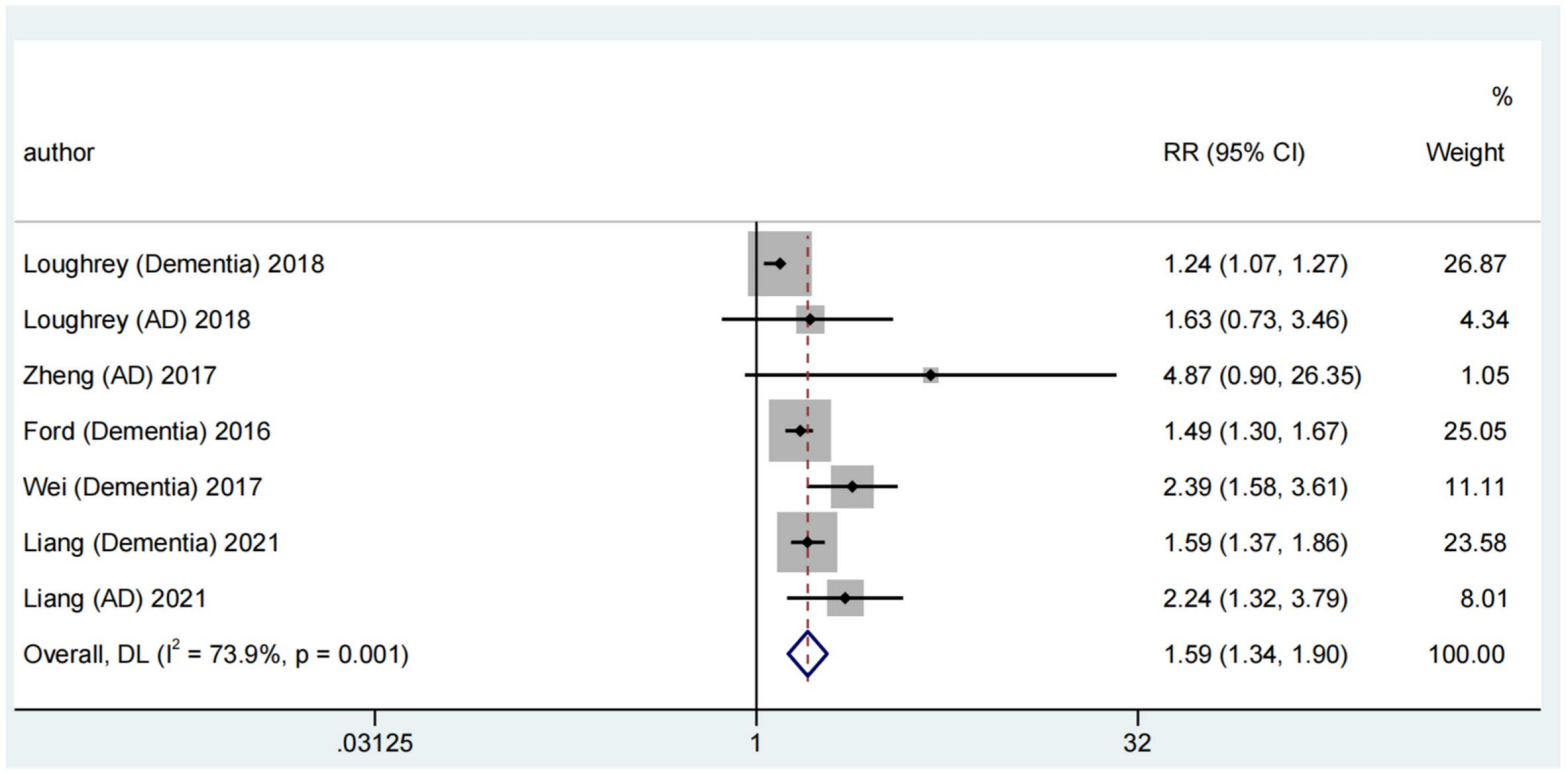
Forest plot of the association between ARHL and risk of dementia.

Considering Alzheimer’s disease as a common dementia type, we conducted a pooled meta-analysis of their association. Among six reviews (Wei et al., 2017; Zheng et al., 2017; Ford et al., 2018; Loughrey et al., 2018; Liang et al., 2021; Kwok et al., 2022) reporting the association of ARHL with dementia and Alzheimer’s disease, five contributed to a pooled meta-analysis (Wei et al., 2017; Zheng et al., 2017; Ford et al., 2018; Loughrey et al., 2018; Liang et al., 2021). This analysis revealed a significant association between ARHL and the risk of dementia and Alzheimer’s disease (RR = 1.59; random-effects; 95% CI: 1.34–1.90; *p* = 0.001; *I*^2^ = 73.9%). The forest plot (Figure 4) illustrates the results of the combined meta-analysis for the association of ARHL with dementia and Alzheimer’s disease. One meta-analysis (Loughrey et al., 2018) reported a significant ARHL–dementia association in cross-sectional and cohort studies; however, no significant association was identified with Alzheimer’s disease. Another meta-analysis (Zheng et al., 2017) showed a relative risk of 4.87 (95% CI: 0.90–26.35; *p* = 0.066) for Alzheimer’s disease in patients with hearing impairment; however, it was insignificant. A more recent meta-analysis (Liang et al., 2021) of prospective cohort studies identified a higher risk of Alzheimer’s disease associated with hearing loss (HR = 2.24; 95% CI: 1.32–3.79; *p* = 0.003).

## 4. Discussion

This comprehensive umbrella review synthesized existing systematic reviews and meta-analyses, assessed their methodological quality and evidence strength, and conducted a pooled meta-analysis of relevant data. With the methodological quality of the studies included in this umbrella review being moderate and the evidence quality being low, the results of the pooled meta-analysis still suggested a strong association of ARHL with cognitive impairment and dementia. Therefore, a thorough examination and understanding of this association holds positive implications for diagnosing and preventing hearing-related health issues and neurodegeneration in the older adult population and is critical for public health decision-making in related domains. Within the older adult demographic presenting hearing abnormalities within the realm of audiology, routine cognitive assessments could aid in the early identification of neurodegeneration. Likewise, individuals with mild cognitive impairment could greatly benefit from timely audiological evaluations. Early identification of risk factors, risk reduction strategies, pathophysiological features, and interventions during the pre-clinical phase of the disease carry substantial advantages for patients, society, and public health at large (Crous-Bou et al., 2017).

Although the precise mechanism underlying the association between hearing impairment and cognitive decline remains unclear, this study does not address the question. However, it is noteworthy that we incorporated a meta-analysis (Yuan et al., 2018) demonstrating that older adults with peripheral and central hearing loss face elevated risks of cognitive impairment compared to those with normal hearing. Moreover, ARHL stemming from cochlear hair cell loss, resulting in reduced auditory cortex, and ARHL due to specific deficits in auditory information processing by the central auditory nerve are associated with cognitive deficits. This association is because aging could lead to hair cell loss and deficits in auditory information processing (Gates et al., 2011; Panza et al., 2018). “Information-degradation” and “common-cause” hypotheses offer credible explanations. The “information-degradation” hypothesis proposes that cognitive decline in older adults results from compensatory mechanisms due to impaired auditory input processing. The cognitive resources are diverted to compensate for sensory impairment, ultimately contributing to cognitive decline (Tun et al., 2009). This hypothesis explains the independent impact of sensory and perceptual impairment on cognitive decline. The “common-cause” hypothesis proposes that a shared mechanism underlies age-related changes in cognition, hearing, and other senses due to widespread neurodegeneration. Both hearing loss and cognitive impairment are considered outcomes of neurodegenerative processes prevalent in the aging brain (Schubert et al., 2017). Hearing loss and neuropathology-induced cognitive impairment co-occur in this hypothesis, explaining the sensory impairments frequently accompanying cognitive decline. No single theory comprehensively explains all the intricacies, indicating that multiple mechanisms could coexist.

The Lancet Society for Prevention, Intervention, and Care has introduced a novel lifetime-based dementia risk model where hearing loss is the most substantial modifiable risk factor among the 12 health and lifestyle factors associated with dementia (Livingston et al., 2020). Utilizing diverse strategies to provide timely and effective medical interventions related to hearing and cognition for individuals with ARHL and cognitive impairment could potentially thwart and slow the onset of dementia while enhancing patients’ quality of life. Hearing aids represent the primary avenue for hearing rehabilitation and enhancing auditory communication capabilities for patients with ARHL (Dawes et al., 2015). Hearing-amplification technological aid could reduce hearing impairment and tinnitus while improving cognitive function, social interaction, and quality of life for individuals with ARHL (Manchaiah et al., 2017). In cases of severe hearing loss, hearing aids might not effectively amplify sound, particularly in higher frequency ranges, possibly leading to partial patient neglect of their condition, contributing to heightened cognitive impairment and dementia. Profound hearing loss necessitates the implementation of a cochlear implant, which converts and integrates external sound signals for transmission to the cochlear nerve (Naples and Ruckenstein, 2020). Despite their benefits in addressing ARHL and cognitive impairment, hearing aids and cochlear implants have limitations. Hearing aids are less effective for severe hearing loss and noisy environments, and cochlear implants are associated with progressive residual hearing loss, expense, and limited availability.

Several limitations exist within our study. Primarily, the GRADE evaluation system, commonly used for assessing evidence quality grades, initially categorized observational studies as low quality, which might impact our studies’ evidence quality grade and recommendation strength. Secondly, inconsistencies in the systematic evaluation of intervention and outcome indicators, and statistical approaches hindered the inclusion of all variables in our analysis, possibly introducing publication bias.

## 5. Conclusion

In conclusion, this comprehensive umbrella review underscores the significant association of ARHL with cognitive impairment and dementia. Hearing loss could pose a substantial risk for cognitive impairment and dementia among older adults. Future intervention studies must validate the effectiveness of earing amplification techniques or related therapies in alleviating ARHL-induced cognitive impairment. Moreover, exploring the causal relationship between ARHL and cognitive impairment remains crucial for devising future accurate diagnosis, prevention, and treatment strategies.

## Data availability statement

The original contributions presented in the study are included in the article/Supplementary material, further inquiries can be directed to the corresponding authors.

## Author contributions

GZ and GY presented this review question and study design, responsible for writing the manuscript, performed the literature screening, data extraction, and quality assessment. XPX screened the potential literature. XX and SS proofread the extracted data. SS participated in the review of the manuscript. All authors contributed to the article and approved the submitted version.

## Funding

This work was supported by Heilongjiang Education Department Young Innovative Talents Training Program (no. UNPYSCT-2020231) and Heilongjiang Province Postdoctoral Research Fund (no. LBH-Q21182).

## Conflict of interest

The authors declare that the research was conducted in the absence of any commercial or financial relationships that could be construed as a potential conflict of interest.

## Publisher’s note

All claims expressed in this article are solely those of the authors and do not necessarily represent those of their affiliated organizations, or those of the publisher, the editors and the reviewers. Any product that may be evaluated in this article, or claim that may be made by its manufacturer, is not guaranteed or endorsed by the publisher.
