# Supplementary material for: Association of age-related hearing loss with cognitive impairment and dementia: an umbrella review
Source: Front Aging Neurosci. 2023 Sep 18;15:1241224. doi: 10.3389/fnagi.2023.1241224 (PMC10543744; doi:10.3389/fnagi.2023.1241224)
Supplement: Supplementary file 1 [file Data_Sheet_1.docx]

**Supplementary material**

**Association of age-related hearing loss with cognitive impairment and dementia：an umbrella review**

1. The definitions of cognitive impairment, dementia, and Alzheimer’s disease
2. Results of the assessment of AMSTAR-2
3. Results of the assessment of GRADE

**The definitions of cognitive impairment, dementia, and Alzheimer disease**

| Dementia  An acquired organic mental disorder with loss of intellectual abilities of sufficient severity to interfere with social or occupational functioning. The dysfunction is multifaceted and involves memory, behavior, personality, judgment, attention, spatial relations, language, abstract thought, and other executive functions. The intellectual decline is usually progressive, and initially spares the level of consciousness.  https://www.ncbi.nlm.nih.gov/mesh/68003704 |
| --- |
| Alzheimer Disease  A degenerative disease of the BRAIN characterized by the insidious onset of DEMENTIA. Impairment of MEMORY, judgment, attention span, and problem solving skills are followed by severe APRAXIAS and a global loss of cognitive abilities. The condition primarily occurs after age 60, and is marked pathologically by severe cortical atrophy and the triad of SENILE PLAQUES; NEUROFIBRILLARY TANGLES; and NEUROPIL THREADS. (From Adams et al., Principles of Neurology, 6th ed, pp1049-57)  https://www.ncbi.nlm.nih.gov/mesh/68000544 |
| Cognitive impairment  Disorders characterized by disturbances in mental processes related to learning, thinking, reasoning, and judgment.  https://www.ncbi.nlm.nih.gov/mesh/68003072 |

**Results of the assessment of AMSTAR-2**

| Included studies | AMSTAR-2 | | | | | | | | | | | | | | | | Overall quality |
| --- | --- | --- | --- | --- | --- | --- | --- | --- | --- | --- | --- | --- | --- | --- | --- | --- | --- |
|  | Q1 | Q2 | Q3 | Q4 | Q5 | Q6 | Q7 | Q8 | Q9 | Q10 | Q11 | Q12 | Q13 | Q14 | Q15 | Q16 |  |
| Lau et al. [21] | Y | Y | Y | PY | Y | Y | Y | Y | Y | Y | Y | N | Y | Y | N | N | M |
| Loughrey et al. [22] | Y | PY | Y | PY | N | Y | PY | Y | Y | Y | Y | Y | Y | Y | N | Y | M |
| Zheng et al. [23] | Y | Y | Y | PY | Y | Y | PY | Y | Y | Y | Y | Y | Y | Y | N | Y | M |
| Ford et al. [24] | Y | N | Y | PY | N | Y | PY | N | N | Y | Y | Y | Y | Y | Y | Y | M |
| Thomson et al. [25] | Y | N | Y | N | N | N | N | Y | N | Y | / | / | Y | Y | N | N | VL |
| Utoomprurkporn et al. [26] | Y | Y | Y | PY | Y | Y | PY | Y | Y | Y | Y | Y | Y | Y | N | Y | M |
| Völter et al. [27] | Y | N | Y | PY | Y | Y | PY | N | N | Y | / | / | N | Y | N | Y | VL |
| Kwok et al. [28] | Y | Y | Y | PY | Y | Y | N | Y | Y | Y | Y | Y | N | Y | N | Y | L |
| Yuan et al. [29] | Y | PY | Y | PY | Y | Y | PY | Y | Y | Y | Y | Y | Y | Y | N | Y | M |
| Wei et al. [30] | Y | N | Y | PY | Y | Y | PY | Y | Y | Y | Y | Y | N | Y | N | Y | M |
| Liang et al. [31] | Y | PY | Y | PY | Y | Y | PY | Y | Y | Y | Y | Y | Y | Y | Y | Y | H |

H, High; M, Moderate; L, Low; VL, Very Low; Y: Yes; PY: Partial Yes; N: No.

**Results of the assessment of GRADE**

| Studies | Design/  Outcomes | Studies  (subjects) | Downgrade factors | | | | | Upgrade factors | | | Evidence quality |
| --- | --- | --- | --- | --- | --- | --- | --- | --- | --- | --- | --- |
|  |  |  | Risk of bias | Inconsistency | Indirectness | Imprecision | Publication  bias | Large effect | Plausible confounding would to change the effect | Dose-response gradients |  |
| Lau et al. [21] | CS/CI | 4(2782) | 0 | 0 | 0 | 0 | -1 | 1 | 0 | 0 | L |
| Loughrey et al. [22] | CS/CI | 5(6553) | 0 | -1 | 0 | 0 | 0 | 1 | 0 | 0 | L |
|  | CO/CI | 3(6825) | 0 | 0 | 0 | 0 | 0 | 0 | 0 | 0 | L |
|  | CS/Dementia + AD | 2(679) | 0 | 0 | 0 | 0 | 0 | 0 | 0 | 0 | L |
|  | CO/Dementia | 1(245) | -1 | 0 | 0 | 0 | -1 | 0 | 0 | 0 | VL |
|  | CO/AD | 1(434) | -1 | -1 | 0 | -1 | -1 | 0 | 0 | 0 | VL |
| Zheng et al. [23] | CO/AD | 3(5477) | 0 | -1 | 0 | -1 | 0 | 1 | 0 | 0 | VL |
|  | CO/CI+AD | 4(7461) | 0 | -1 | 0 | 0 | 0 | 1 | 0 | 0 | L |
| Ford et al. [24] | CO/Dementia | 13(72831) | 0 | 0 | 0 | 0 | 0 | 0 | 0 | 0 | L |
| Thomson et al. [25] | Dementia | 17(1378444) | -1 | 0 | 0 | 0 | -1 | 0 | 0 | 0 | VL |
| Utoomprurkporn et al. [26] | NS/CI(MoCA) | 4(533) | 0 | -1 | 0 | 0 | 0 | 0 | 0 | 0 | VL |
| Völter et al. [27] | NS/CI(MMSE, MoCA) | 4(425) | -1 | 0 | 0 | 0 | -1 | 0 | 0 | 0 | VL |
| Kwok et al. [28] | CO/(0.5-2 kHz PTA） | 5(365) | 0 | 0 | 0 | -1 | 0 | 0 | 0 | 0 | VL |
|  | CO/(0.5-4 kHz PTA） | 6(393) | 0 | 0 | 0 | -1 | 0 | 0 | 0 | 0 | VL |
| Yuan et al. [29] | CO/CI  (follow-up≤6 years) | 3(4926) | 0 | 0 | 0 | 0 | 0 | 0 | 0 | 0 | L |
|  | CO/CI  (follow-up>6 years) | 2(3527) | 0 | 0 | 0 | 0 | 0 | 0 | 0 | 0 | L |
|  | CO/CI  (total) | 4(6815) | 0 | 0 | 0 | 0 | 0 | 0 | 0 | 0 | L |
| Wei et al. [30] | CO/CI | 4(7524) | 0 | 0 | 0 | 0 | 0 | 0 | 0 | 0 | L |
|  | CO/Dementia | 7(10943) | 0 | -1 | 0 | 0 | 0 | 1 | 0 | 0 | L |
| Liang et al. [31] | CO/Dementia | 11(721384) | 0 | -1 | 0 | 0 | 0 | 0 | 0 | 0 | VL |
|  | CO/AD | 5(4630) | 0 | 0 | 0 | 0 | 0 | 1 | 0 | 0 | M |
